# Supplementary material for: Dynamic stability of Sgt2 enables selective and privileged client handover in a chaperone triad
Source: Nat Commun. 2024 Jan 2;15:134. doi: 10.1038/s41467-023-44260-5 (PMC10761869; doi:10.1038/s41467-023-44260-5)
Supplement: Supplementary file 1 — Supplementary Information [file 41467_2023_44260_MOESM1_ESM.pdf]

Supplementary Material for

Dynamic stability of Sgt2 enables selective and privileged client handover in a chaperone triad

Hyunju Cho[1,4], Yumeng Liu[1,5], SangYoon Chung[2], Sowmya Chandrasekar[1], Shimon Weiss[2,3], Shu-ou Shan[1]*

This file includes figures S1-8, figure legends, Table S1, and reference.

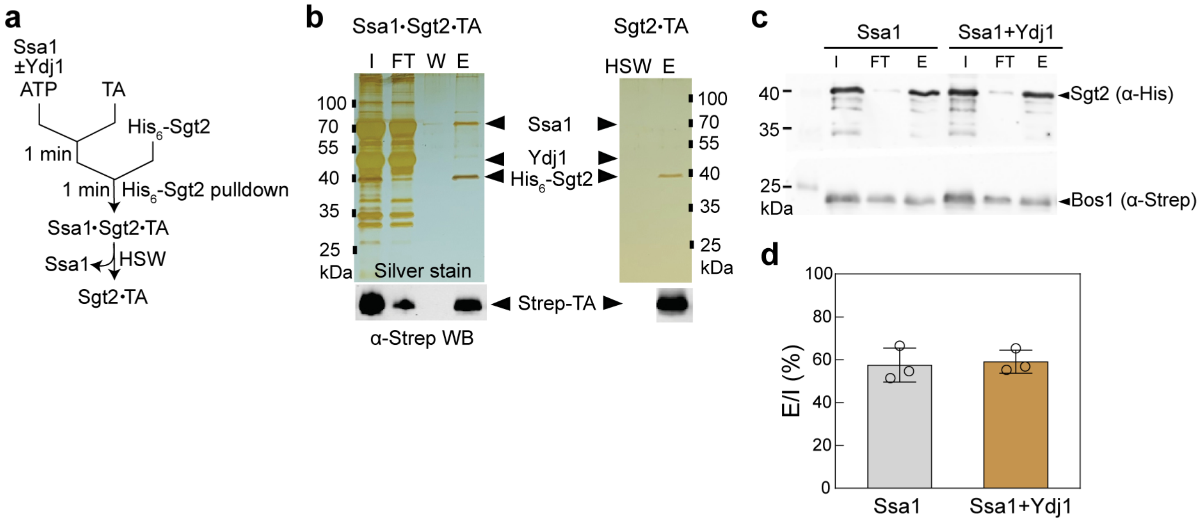

Supplementary Figure 1. Preparation of Ssa1-Sgt2-TA and Sgt2-TA complexes via TA transfer from Ssa1. (**a**) Scheme for purification of the Sgt2-TA complex with or without Ssa1 bound. See SI Methods for details. (**b**) The complexes purified as depicted in (a) were analyzed by SDS-PAGE. Ssa1, Ydj1, and Sgt2 were visualized by silver stain, and the TA substrate was detected by western blot against the Strep tag. I, FT, and E denote total input, flowthrough, and elution, respectively. Data are shown for the complex generated with Ydj1 present during TA transfer. Note that Ydj1 did not co-purify with the Ssa1-Sgt2-TA complex, and the same complex composition was obtained without Ydj1. (**c,d**) Ydj1 does not increase the amount of TA loaded on Sgt2 during the Ssa1-to-Sgt2 transfer. Sgt2-TA complexes were generated with and without Ydj1 and purified with high-salt wash (HSW) as outlined in Fig. S1A and described in the Methods. The amount of TA bound to Sgt2 was analyzed by western blot against strep-tagged TA and His_6-tagged Sgt2 (c), and quantified in (d). Values represent mean ± SD, with n = 3. Source data are provided as a Source Data file.

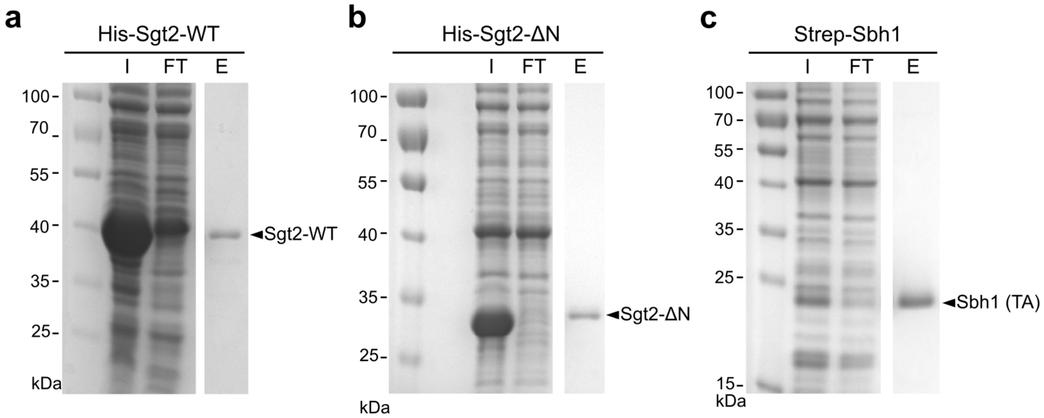

Supplementary Figure 2. Purification of WT His$_6$-Sgt2 (a), His$_6$-Sgt2ΔN (b), and Strep-Sbh1 (c), analyzed by SDS-PAGE and coomassie-blue staining. Source data are provided as a Source Data file.

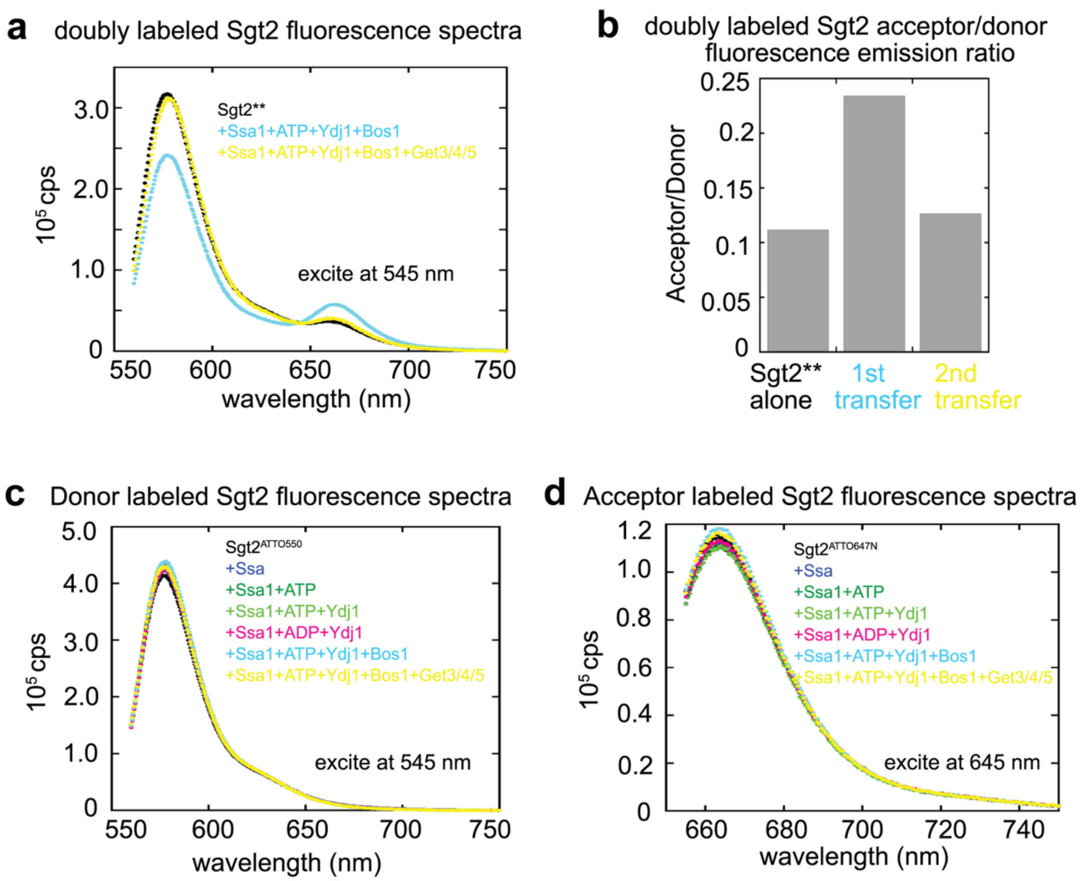

Supplementary Figure 3. Bulk fluorescence data for doubly labeled Sgt2 and controls for dye photophysics. (**a**) Fluorescence emission spectra of Sgt2 doubly labeled with donor and acceptor dyes under the following conditions: (i) Sgt2 alone (*black*); (ii) in the Ssa1-Sgt2-TA complex after the first TA transfer (*blue*); (iii) after the second TA transfer from Sgt2 to Get3 in the presence of Get4/5 (*orange*). Samples were prepared as in Figure 3, except that 15 nM doubly labeled Sgt2 was used. cps, counts per second. **(b**) Fluorescence intensity ratio of the acceptor and donor dyes is quantified from the data in (**a**). **(c, d)** Fluorescence emission spectra of Sgt2 singly labeled with the donor (**c**) or acceptor (**d**) dye in the presence of the indicated ligands and binding partners. Protein concentrations are the same as those in Figure 3.

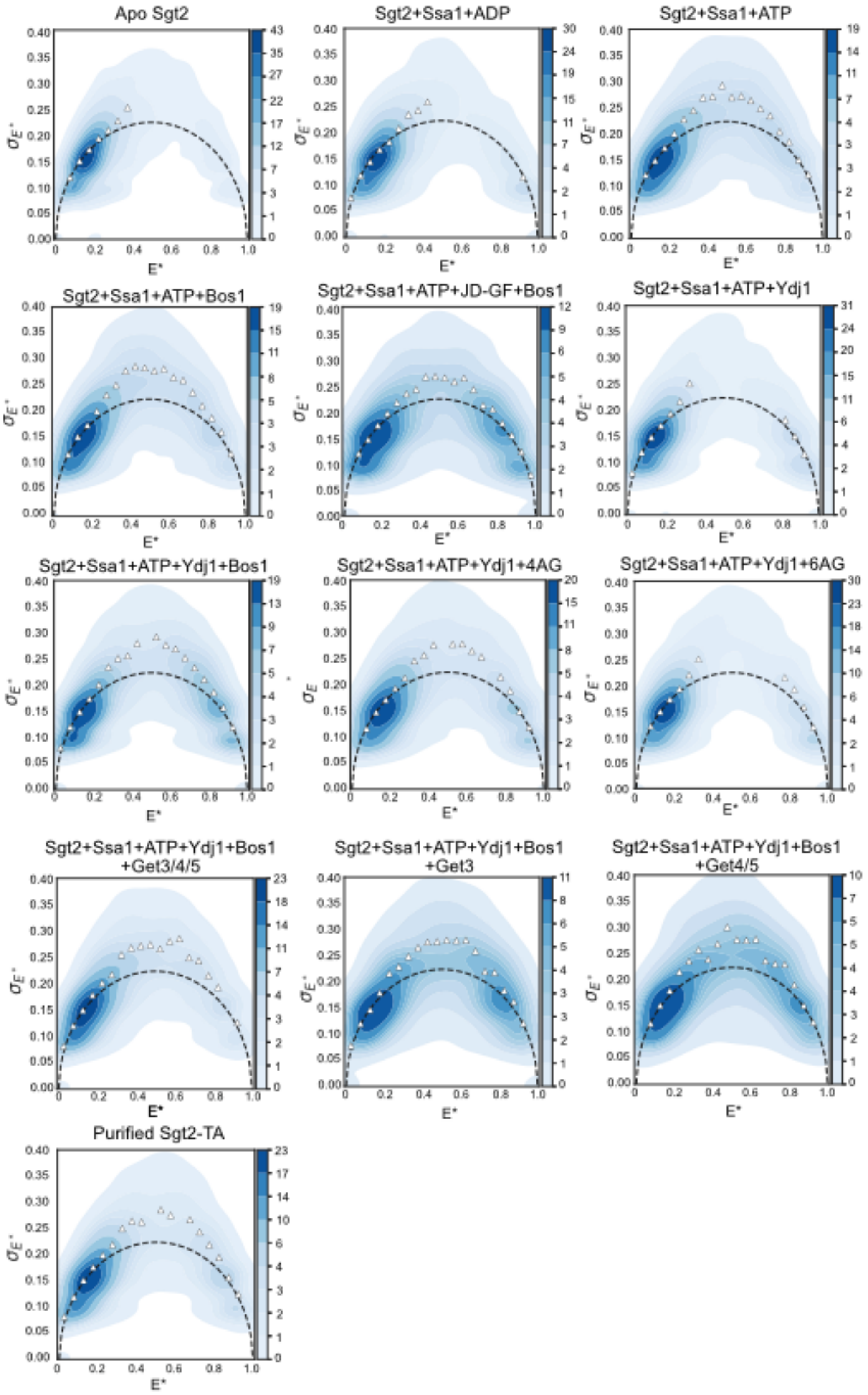
Apo Sgt2
Sgt2+Ssa1+ADP
Sgt2+Ssa1+ATP
Sgt2+Ssa1+ATP+Bos1
Sgt2+Ssa1+ATP+JD-GF+Bos1
Sgt2+Ssa1+ATP+Ydj1
Sgt2+Ssa1+ATP+Ydj1+Bos1
Sgt2+Ssa1+ATP+Ydj1+4AG
Sgt2+Ssa1+ATP+Ydj1+6AG
Sgt2+Ssa1+ATP+Ydj1+Bos1 +Get3/4/5
Sgt2+Ssa1+ATP+Ydj1+Bos1 +Get3
Sgt2+Ssa1+ATP+Ydj1+Bos1 +Get4/5
Purified Sgt2-TA
E*
σ_{E^*}

Supplementary Figure 4. **Burst variance analysis (BVA) of the dynamics of conformational sampling of Sgt2 under the indicated conditions**. Triangles denote the mean SD for the individual FRET bins; dashed lines show the shot-noise limited SD.

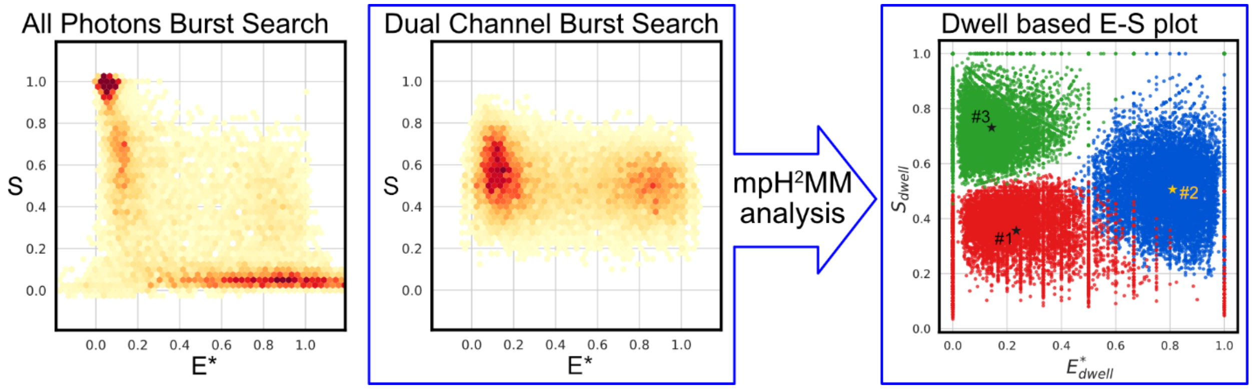

Supplementary Figure 5. µS-ALEX data analysis work-flow. *Middle panel,* burst-based E-S histogram after dual-channel burst-search (DCBS). The signals of fluorescent molecules (bursts) were identified and collected using DCBS and burst selection as described in Methods. DCBS eliminates donor-only and acceptor-only species and can also mitigate artifacts caused by dye-photophysical effects such as bleaching. *Right panel,* dwell-based E-S plot after multi-parameter photon-by-photon hidden Markov modeling (mpH^{2}MM) analysis on the bursts identified by DCBS. The Viterbi algorithm in mpH^{2}MM identifies the most likely state path and corresponding transitions kinetics within each burst. Each identified state within a burst was considered a dwell. *Left panel,* burst-based E-S histogram after all photon burst-search and a burst selection of $n_D^D + n_D^A + n_A^A \geq 100$, shown for comparison with the results after DCBS (middle panel).

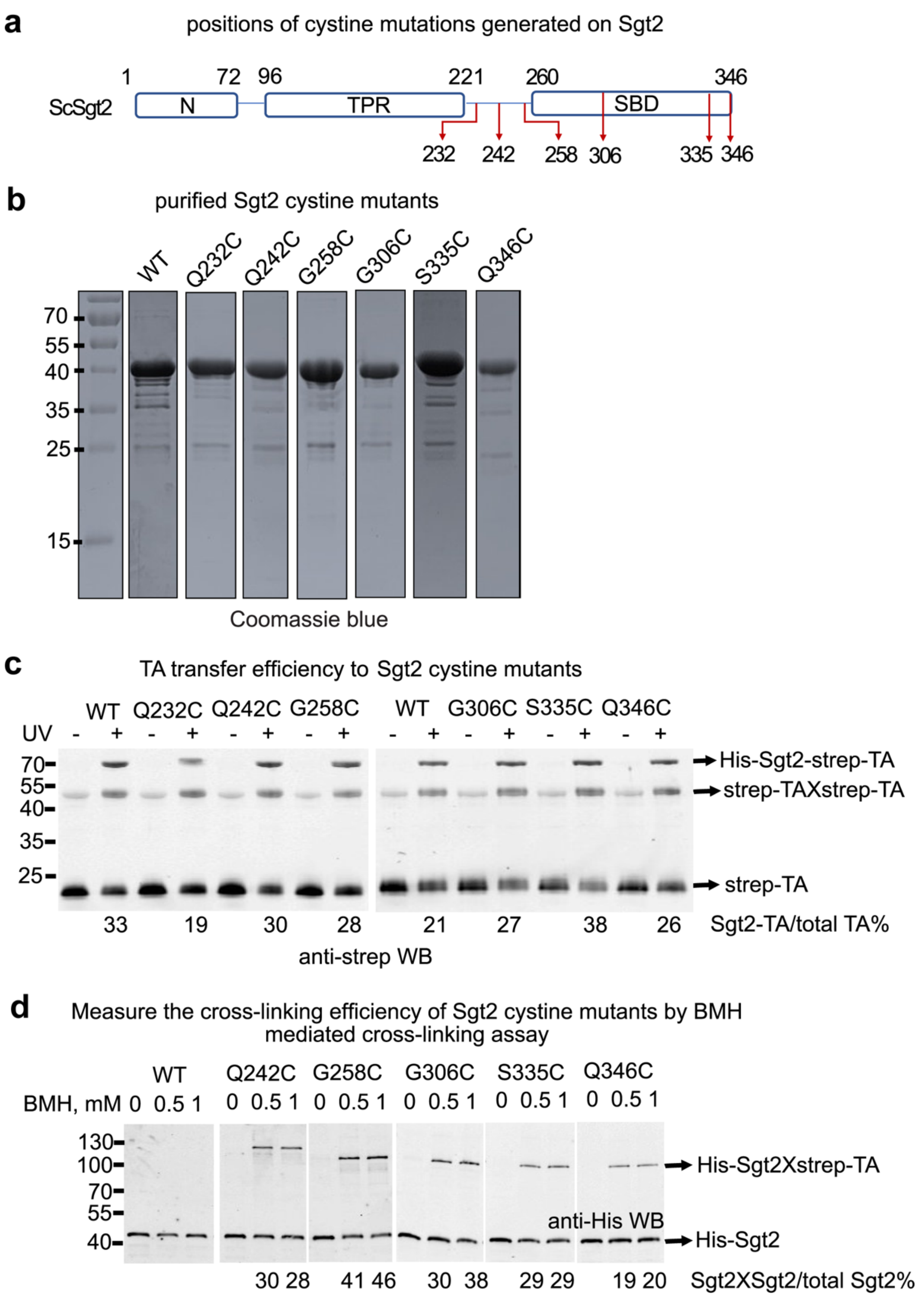

Supplementary Figure 6. Characterization of Sgt2 single cysteine mutants. (a) Scheme of Sgt2 showing the sites of cysteine mutations. (**b**) Coomassie-blue stained SDS-PAGE for purified Sgt2 single cysteine mutants. (**c**) Ssa1-to-Sgt2 TA transfer was measured for single cysteine Sgt2 mutants using crosslinking of Bpa, incorporated at the 8th residue of the TMD of recombinant purified strep-Bos1. Ssa1-to-Sgt2 TA transfer and UV crosslinking were carried out

as described in SI Methods. TA crosslinking to Sgt2 was visualized by western blot against Strep-tagged TA. The percentage of cross-linked Sgt2-TA are indicated. **(d)** Intradimer crosslink of Sgt2 single cysteine mutants by BMH. 0.3 μM His-Sgt2 was incubated with 1 mM BMH at room temperature for 1h. Reactions were visualized by anti-His western blot (for Sgt2). The percentage of cross-linked Sgt2 are indicated.

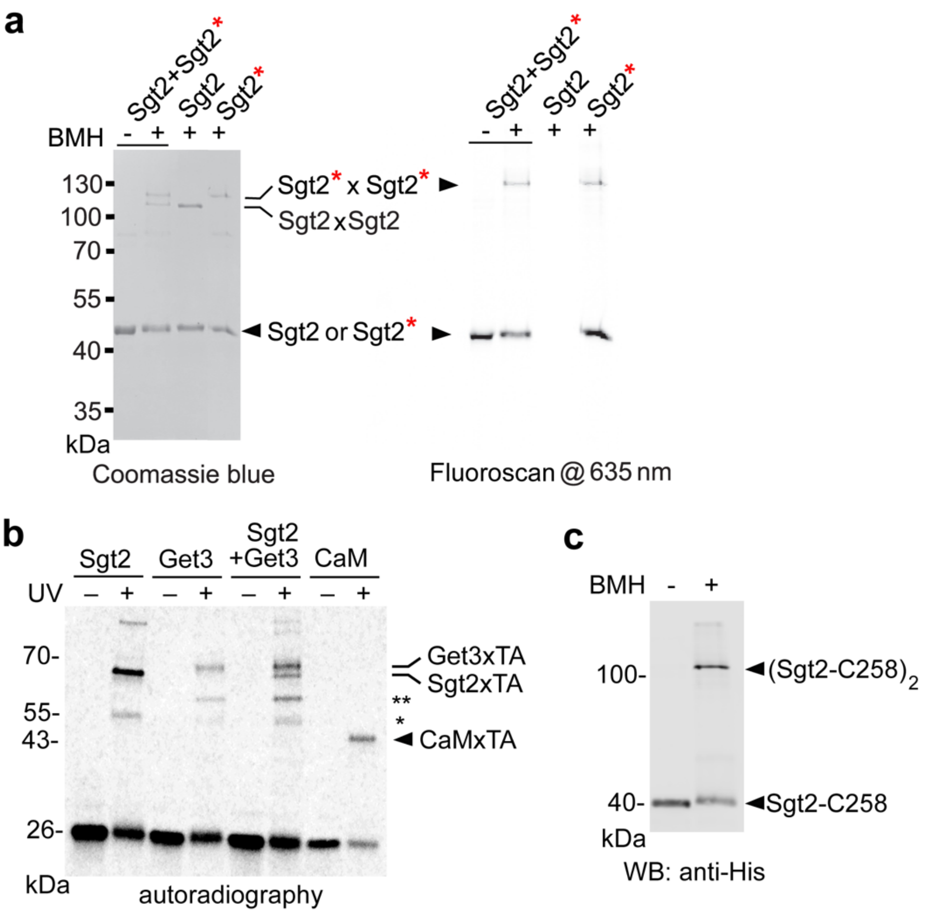

Supplementary Figure 7. Preparation and validation of BMH crosslinked Sgt2-TA complexes. (**a**) BMH-induced intra-dimer crosslink in Sgt2-C258. Sgt2-C258 (abbreviated as Sgt2) was labeled with ATTO647N (Sgt2*). 0.5 μM Sgt2, Sgt2*, or their equimolar mixture was crosslinked with 1 mM BMH at room temperature for 1h. Crosslinked and uncrosslinked proteins were resolved by SDS-PAGE and visualized by Coomassie blue staining and fluoroscan with excitation at 635 nm. Fluorescence labeling resulted in an upward shift of crosslinked Sgt2 (Sgt2xSgt2). The cross-linked product in the Sgt2 + Sgt2* sample corresponds to that of either Sgt2 or Sgt2*, without observation of a species with intermediary mobility expected for Sgt2xSgt2*, indicating the absence of inter-dimer crosslinks or Sgt2 exchange between dimers. (**b**) Autoradiography showing TA^{Bpa} and its UV-induced crosslink to different chaperones. * and ** denote minor crosslinked species in the presence of Sgt2 or Get3 that were not interpreted. (**c**) The purified Sgt2-C258-TA complex was crosslinked with BMH and visualized by western blot against His_6-tagged Sgt2-C258.

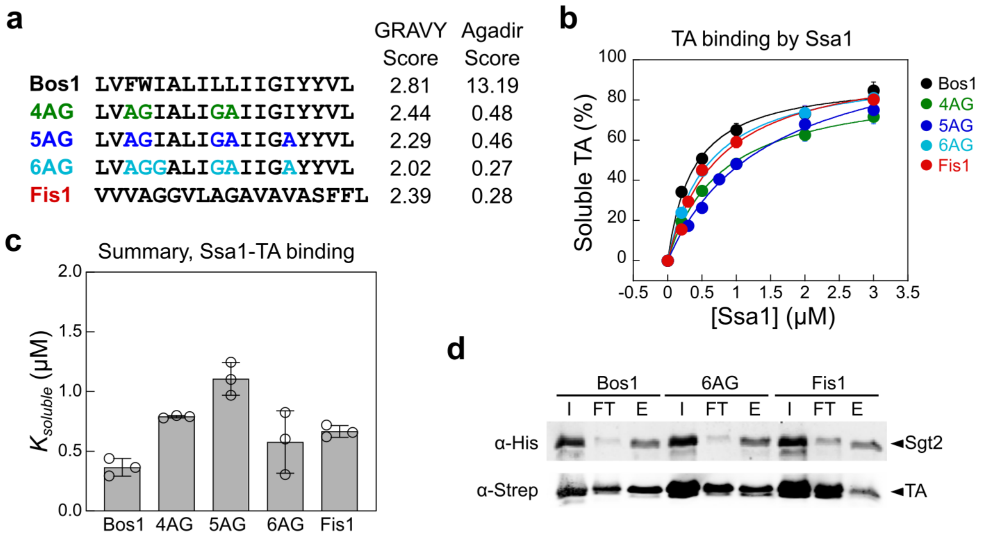

Supplementary Figure 8. Binding of TA variants to Ssa1. (**a**) Sequence of the TMD in the TA variants tested. Grand Average of Hydropathy (GRAVY) scores and Agadir scores (helical content) for the TMD of TA variants are listed (also see reference[1]). **(b, c)** All TA variants were efficiently captured and solubilized by Ssa1. The ability of Ssa1 to capture and solubilize recombinant purified TA variants was measured using the turbidity assay (see SI Methods) (**b**). The data were fit to Eq 5, and the obtained solubilization constants are summarized in (**c**). Values represent mean ± SD, with n = 3. (**d**) The association of TA variants with Sgt2 after transfer from Ssa1. Sgt2-TA complexes were generated and purified with high-salt wash (HSW) as outlined in Fig. S1A. The amount of TA bound to Sgt2 was analyzed by western blot analysis against strep-tagged TA. Source data are provided as a Source Data file.

Name	# of replicates	$k_{open→close}$ (S^{-1})	$k_{close→open}$ (S^{-1})	$K_{closing}$
Apo-Sgt2	8	57.65 ± 7.89	260.93 ± 43.48	0.221 ± 0.048
Sgt2-Bos1 (Purified)	5	261.24 ± 24.91	437.37 ± 64.06	0.597 ± 0.104
+Ssa1^{ATP}	3	305.40 ± 25.55	388.94 ± 42.54	0.785 ± 0.108
+Ssa1^{ADP}	1	76.19 ± 18.10	210.18 ± 74.50	0.362 ± 0.155
+Ssa1^{ATP}+Ydj1	3	90.99 ± 4.87	192.44 ± 27.43	0.473 ± 0.072
+Ssa1^{ATP}+Bos1	7	275.16 ± 30.91	344.73 ± 67.03	0.798 ± 0.179
+Ssa1^{ATP}+Ydj1+Bos1	10	255.33 ± 46.09	272.50 ± 27.77	0.937 ± 0.194
+Ssa1^{ATP}+JD-GF+Bos1	2	319.85 ± 69.36	325.25 ± 40.21	0.983 ± 0.245
+Ssa1^{ATP}+Ydj1+4AG	3	140.38 ± 6.71	194.37 ± 16.05	0.722 ± 0.069
+Ssa1^{ATP}+Ydj1+6AG	10	102.87 ± 9.54	207.16 ± 12.09	0.497 ± 0.054
+Ssa1^{ATP}+Ydj1+Bos1+Get3	2	329.69	372.03	0.886
+Ssa1^{ATP}+Ydj1+Bos1+Get4/5	2	414.31	406.91	1.018
+Ssa1^{ATP}+Ydj1+Bos1+Get3/4/5	4	174.84 ± 54.53	332.78 ± 61.04	0.525 ± 0.190

Supplementary Table 1. Rate and equilibrium constants of Sgt2 conformational distribution and interconversion estimated by mpH^{2}MM analysis. The rate constants of Sgt2 interconversion between open and closed conformations are extracted from mpH^{2}MM analysis shown in Supplementary Dataset 1. The equilibrium of Sgt2 closing is calculated using Eq13. Values are reported as mean ± S.D., estimated by 1) dividing the total bursts into subsets that contains 1700~2000 bursts, 2) performing mpH^{2}MM analysis on the subsets with the 3-state model to extract kinetic constants for each subset, and 3) calculating the standard deviation of the kinetic constants, as described under Methods.

Supplementary References

1. Rao, M. *et al.* Multiple selection filters ensure accurate tail-anchored membrane protein targeting. *eLife* **5**, e21301 (2016).
