# Supplementary material for: Dynamic stability of Sgt2 enables selective and privileged client handover in a chaperone triad
Source: Nat Commun. 2024 Jan 2;15:134. doi: 10.1038/s41467-023-44260-5 (PMC10761869; doi:10.1038/s41467-023-44260-5)
Supplement: Supplementary file 3 — Description of Additional Supplementary Files [file 41467_2023_44260_MOESM3_ESM.pdf]

File Name: Supplementary Dataset 1

Description: mpH2MM analysis of all µs-ALEX data
