# Supplementary material for: Dynamic stability of Sgt2 enables selective and privileged client handover in a chaperone triad
Source: Nat Commun. 2024 Jan 2;15:134. doi: 10.1038/s41467-023-44260-5 (PMC10761869; doi:10.1038/s41467-023-44260-5)
Supplement: Supplementary file 4 — Supplementary Data [file 41467_2023_44260_MOESM4_ESM.pdf]

Apo-Sgt2

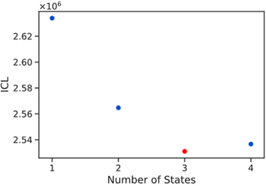

$k_{1\rightarrow2} = 57.65 \pm 7.89$ /sec
$k_{1\rightarrow3} = 1534.26 \pm 133.07$ /sec
$k_{2\rightarrow1} = 260.93 \pm 43.48$ /sec
$k_{2\rightarrow3} = 391.64 \pm 50.37$ /sec
$k_{3\rightarrow1} = 1475.38 \pm 183.54$ /sec
$k_{3\rightarrow2} = 107.96 \pm 16.82$ /sec

3-state model (Selected, ICL minimized)

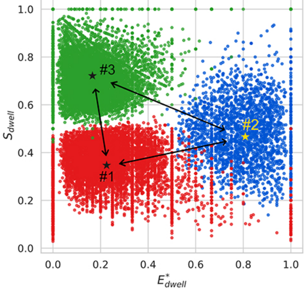

Sgt2-Bos1 (Purified complex)

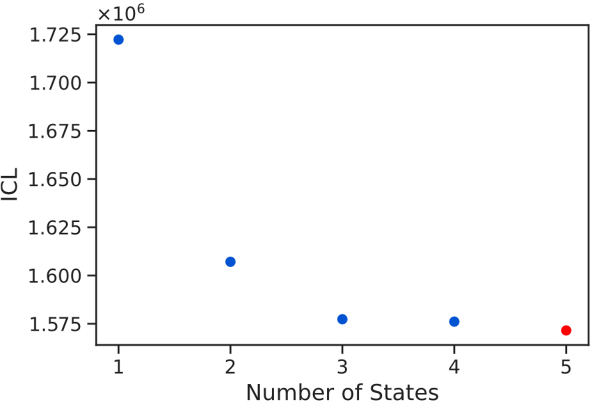

$k_{1\rightarrow2}$ = 261.24 ± 24.91 /sec
$k_{1\rightarrow3}$ = 1800.47 ± 177.88 /sec
$k_{2\rightarrow1}$ = 437.37 ± 64.06 /sec
$k_{2\rightarrow3}$ = 316.91 ± 42.70 /sec
$k_{3\rightarrow1}$ = 1757.48 ± 116.55 /sec
$k_{3\rightarrow2}$ = 203.29 ± 31.95 /sec

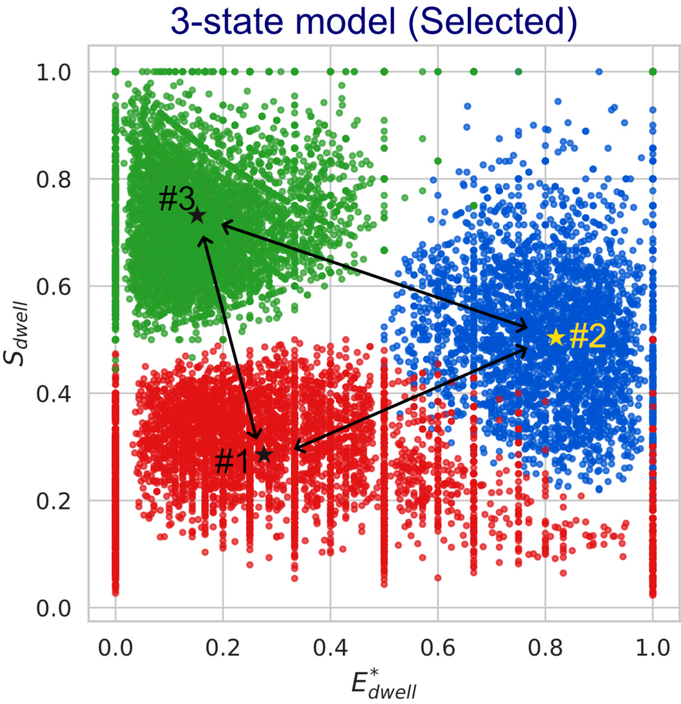

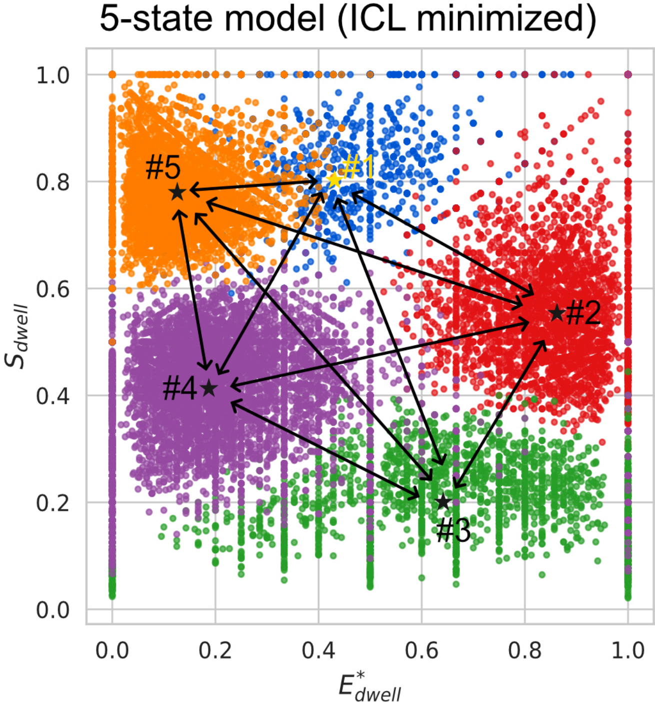

$k_{1\rightarrow2}$ = 3251.83 /s
$k_{1\rightarrow3}$ = 5627.78 /s
$k_{1\rightarrow4}$ = 3924.84 /s
$k_{1\rightarrow5}$ = 219.12 /s
$k_{2\rightarrow1}$ = 1154.92 /s
$k_{2\rightarrow3}$ = 435.56 /s
$k_{2\rightarrow4}$ = 5.5×10^{-116}/s
$k_{2\rightarrow5}$ = 81.22 /s
$k_{3\rightarrow1}$ = 270.31 /s
$k_{3\rightarrow2}$ = 1155.93 /s
$k_{3\rightarrow4}$ = 1148.92 /s
$k_{3\rightarrow5}$ = 2.2×10^{-60} /s
$k_{4\rightarrow1}$ = 357.30 /s
$k_{4\rightarrow2}$ = 1.9×10^{-31} /s
$k_{4\rightarrow3}$ = 216.20 /s
$k_{4\rightarrow5}$ = 1248.79 /s
$k_{5\rightarrow1}$ = 140.78 /s
$k_{5\rightarrow2}$ = 26.80 /s
$k_{5\rightarrow3}$ = 7.3×10^{-101} /s
$k_{5\rightarrow4}$ = 1651.07 /s

+Ssa1^{ATP}

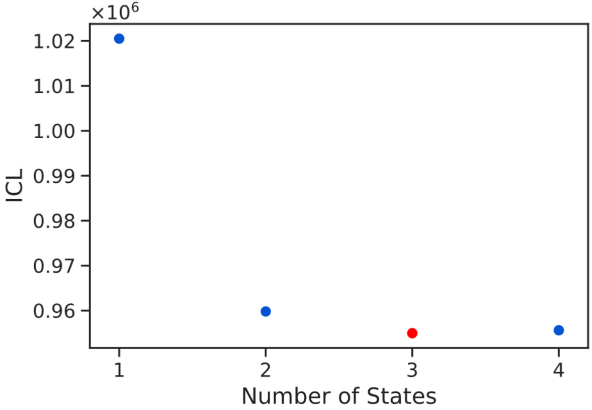

$k_{1\rightarrow2}$ = 305.40 ± 25.55 /sec

$k_{1\rightarrow3}$ = 1623.00 ± 131.88 /sec

$k_{2\rightarrow1}$ = 388.94 ± 42.54 /sec

$k_{2\rightarrow3}$ = 316.99 ± 19.82 /sec

$k_{3\rightarrow1}$ = 1530.15 ± 124.47 /sec

$k_{3\rightarrow2}$ = 301.80 ± 16.91 /sec

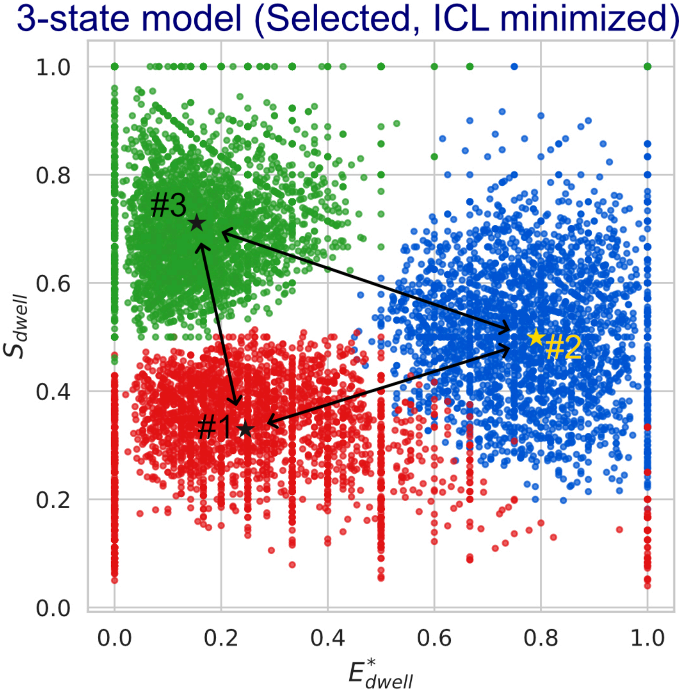

+Ssa1^{ADP}

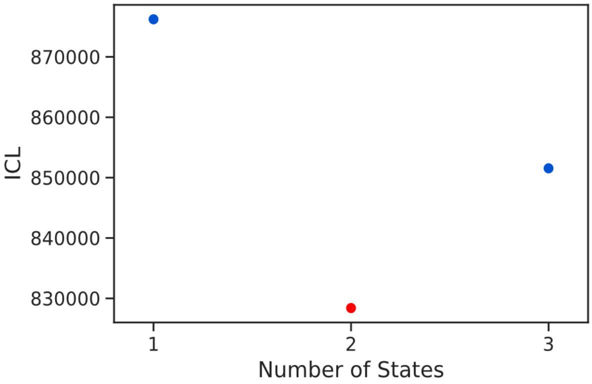

$k_{1\rightarrow2}$ = 76.19 ± 18.10 /sec
$k_{1\rightarrow3}$ = 2081.69 ± 72.48 /sec
$k_{2\rightarrow1}$ = 210.18 ± 74.50 /sec
$k_{2\rightarrow3}$ = 196.68 ± 50.63 /sec
$k_{3\rightarrow1}$ = 2275.59 ± 143.92 /sec
$k_{3\rightarrow2}$ = 84.69 ± 18.85 /sec

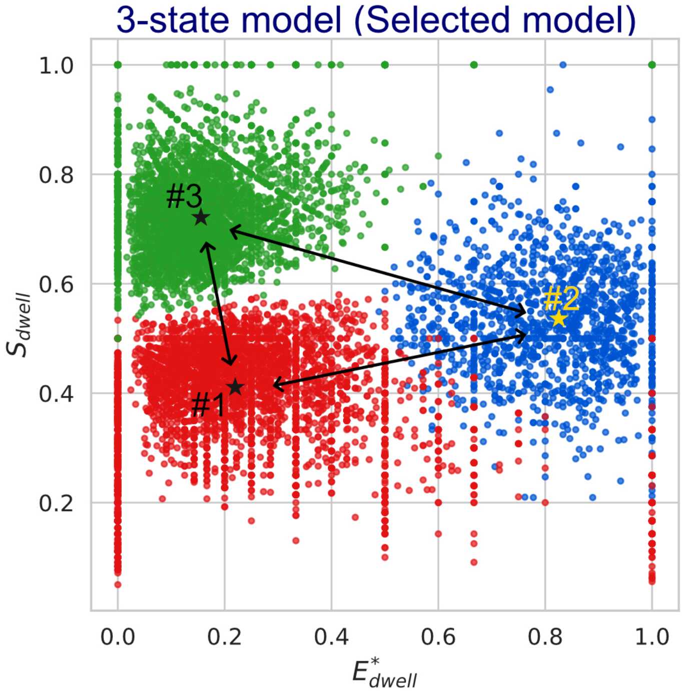

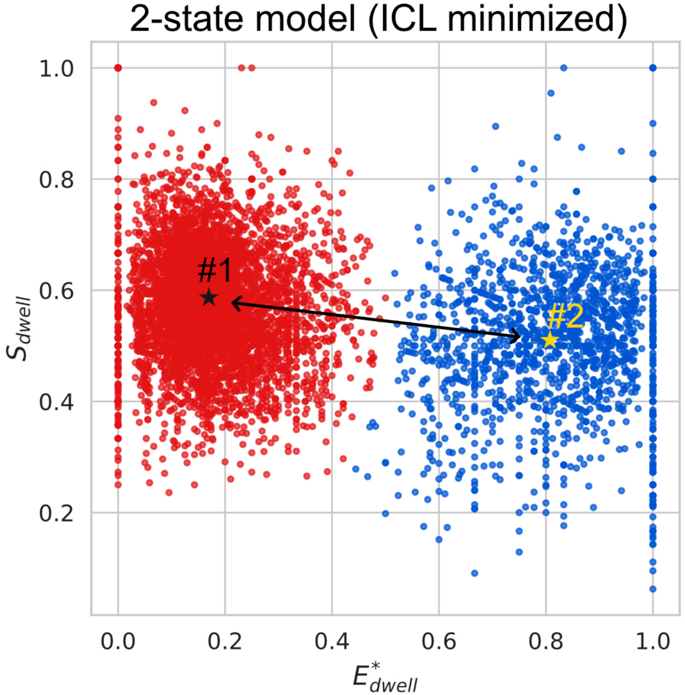

$k_{1\rightarrow2}$ = 115.57 /sec
$k_{2\rightarrow1}$ = 449.99 /sec

+Ssa1^{ATP}+Ydj1

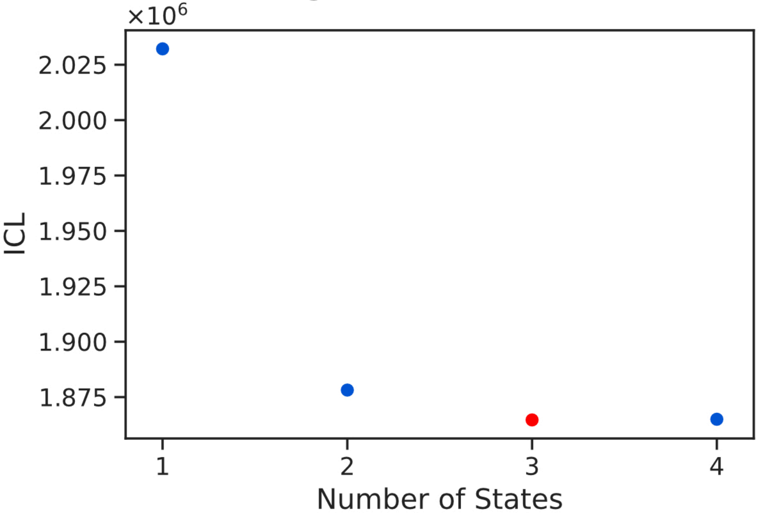

$k_{1\rightarrow2}$ = 90.99 ± 4.87 /sec
$k_{1\rightarrow3}$ = 1014.30 ± 96.50 /sec
$k_{2\rightarrow1}$ = 192.44 ± 27.43 /sec
$k_{2\rightarrow3}$ = 192.94 ± 22.33 /sec
$k_{3\rightarrow1}$ = 1348.50 ± 92.54 /sec
$k_{3\rightarrow2}$ = 107.33 ± 18.38 /sec

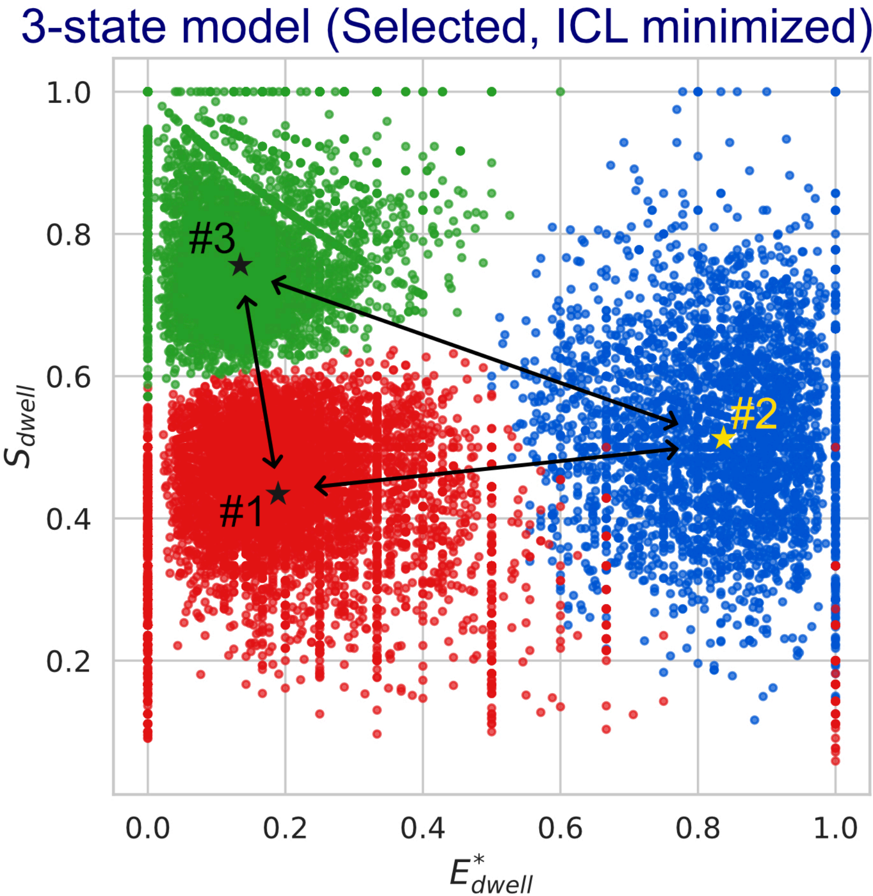

+Ssa1^{ATP}+Bos1

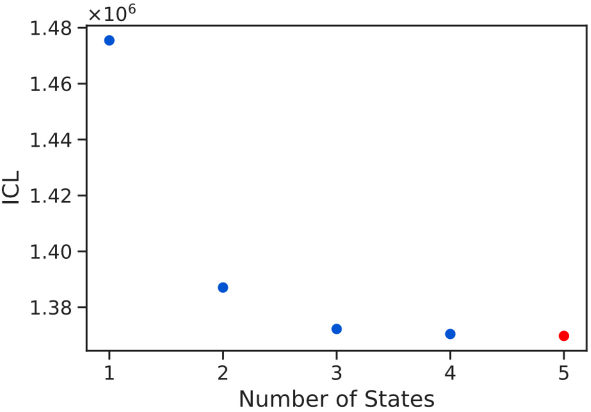

$k_{1\to2}$ = 275.16 ± 30.91 /sec
$k_{1\to3}$ = 1861.48 ± 126.17 /sec
$k_{2\to1}$ = 344.73 ± 67.03 /sec
$k_{2\to3}$ = 371.39 ± 45.78 /sec
$k_{3\to1}$ = 1584.17 ± 181.18 /sec
$k_{3\to2}$ = 271.58 ± 35.26 /sec

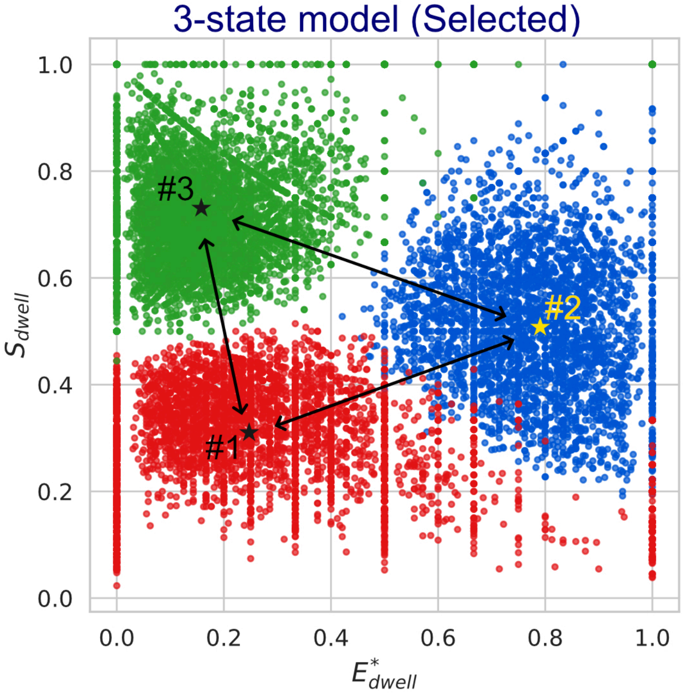

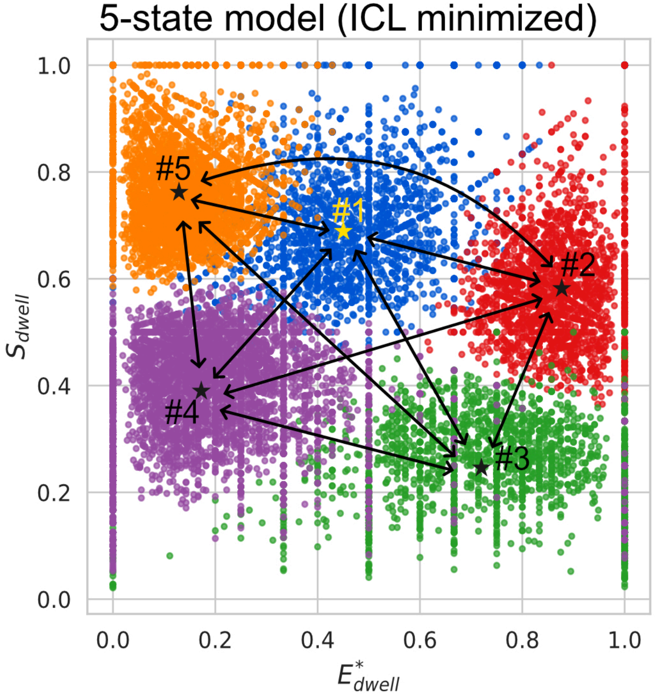

$k_{1\to2}$ = 969.24 /s
$k_{1\to3}$ = 2410.04 /s
$k_{1\to4}$ = 577.38 /s
$k_{1\to5}$ = 424.66 /s
$k_{2\to1}$ = 990.33 /s
$k_{2\to3}$ = 565.80 /s
$k_{2\to4}$ = 5.8×10^{-30} /s
$k_{2\to5}$ = 57.51 /s
$k_{3\to1}$ = 1349.79 /s
$k_{3\to2}$ = 907.29 /s

$k_{3\to4}$ = 539.78 /s
$k_{3\to5}$ = 5.4×10^{-6} /s
$k_{4\to1}$ = 115.20 /s
$k_{4\to2}$ = 1.5×10^{-12} /s
$k_{4\to3}$ = 180.85 /s
$k_{4\to5}$ = 1256.43 /s
$k_{5\to1}$ = 188.84 /s
$k_{5\to2}$ = 12.33 /s
$k_{5\to3}$ = 4.5×10^{-31} /s
$k_{5\to4}$ = 1390.37 /s

+Ssa1^{ATP}+Ydj1+Bos1

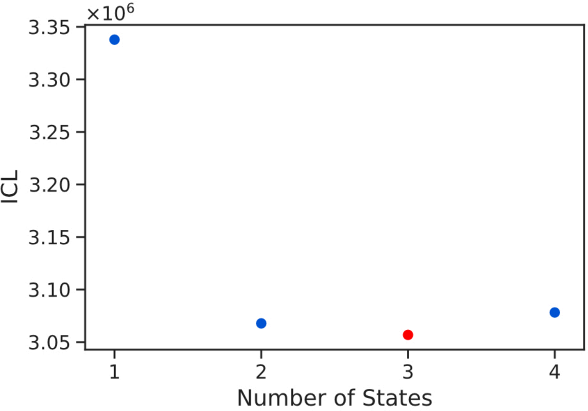

$k_{1\to2}$ = 255.33 ± 46.09 /sec
$k_{1\to3}$ = 1901.13 ± 244.30 /sec
$k_{2\to1}$ = 272.50 ± 27.77 /sec
$k_{2\to3}$ = 237.35 ± 29.97 /sec
$k_{3\to1}$ = 2022.68 ± 186.19 /sec
$k_{3\to2}$ = 288.99 ± 36.50 /sec

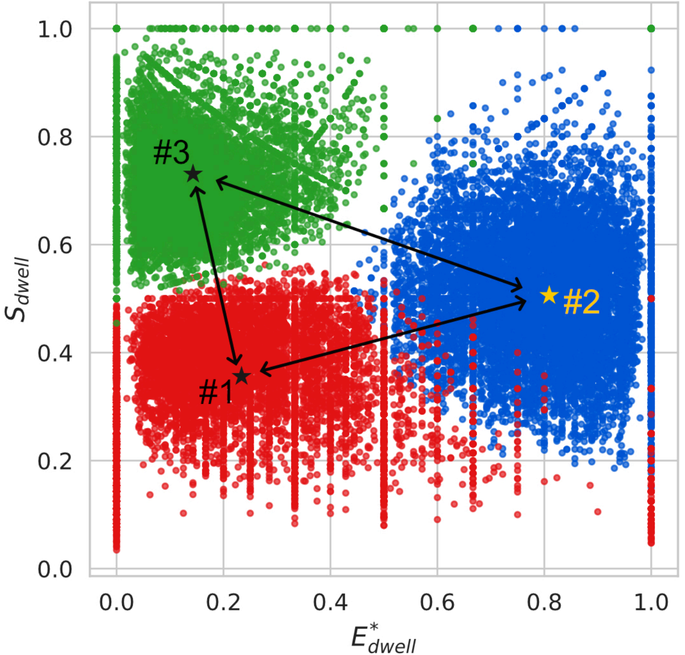

+Ssa1^{ATP}+JD-GF+Bos1

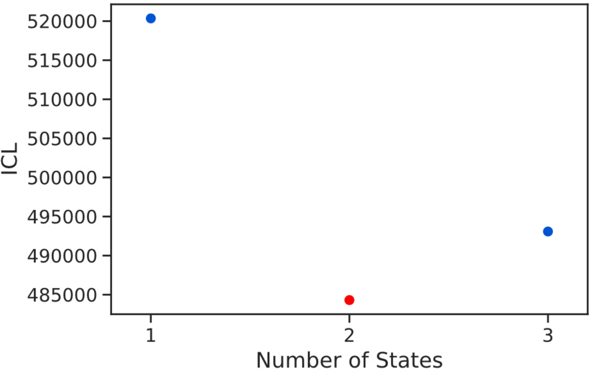

$k_{1\rightarrow2}$ = 319.85 ± 69.36 /sec
$k_{1\rightarrow3}$ = 3469.68 ± 53.23 /sec
$k_{2\rightarrow1}$ = 325.25 ± 40.21 /sec
$k_{2\rightarrow3}$ = 205.68 ± 59.46 /sec
$k_{3\rightarrow1}$ = 3338.04 ± 94.73 /sec
$k_{3\rightarrow2}$ = 281.57 ± 101.73 /sec

3-state model (Selected)

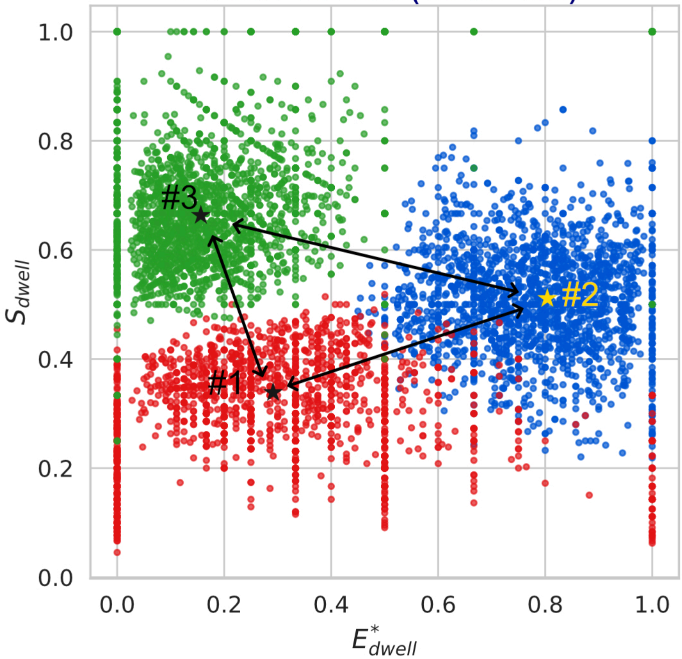

2-state model (ICL minimized)

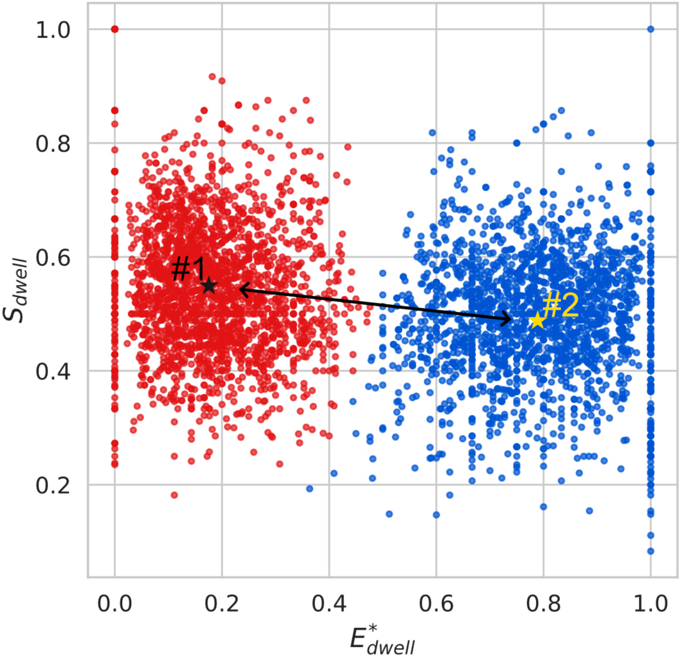

$k_{1\rightarrow2}$ = 387.55 /sec
$k_{2\rightarrow1}$ = 465.47 /sec

+Ssa1^{ATP}+Ydj1+4AG

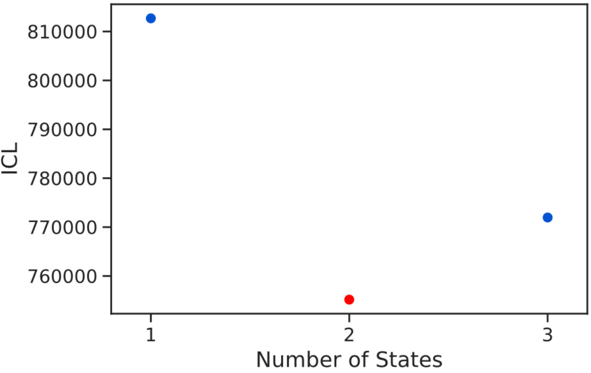

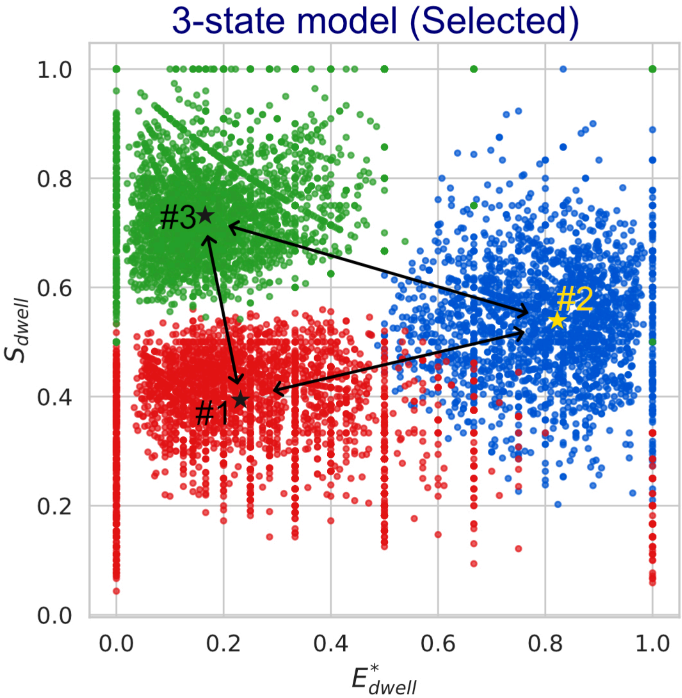

$k_{1\to2}$ = 140.38 ± 6.71 /sec
$k_{1\to3}$ = 2409.99 ± 162.30 /sec
$k_{2\to1}$ = 194.37 ± 16.05 /sec
$k_{2\to3}$ = 226.41 ± 26.49 /sec
$k_{3\to1}$ = 2589.97 ± 27.24 /sec
$k_{3\to2}$ = 194.24 ± 27.44 /sec

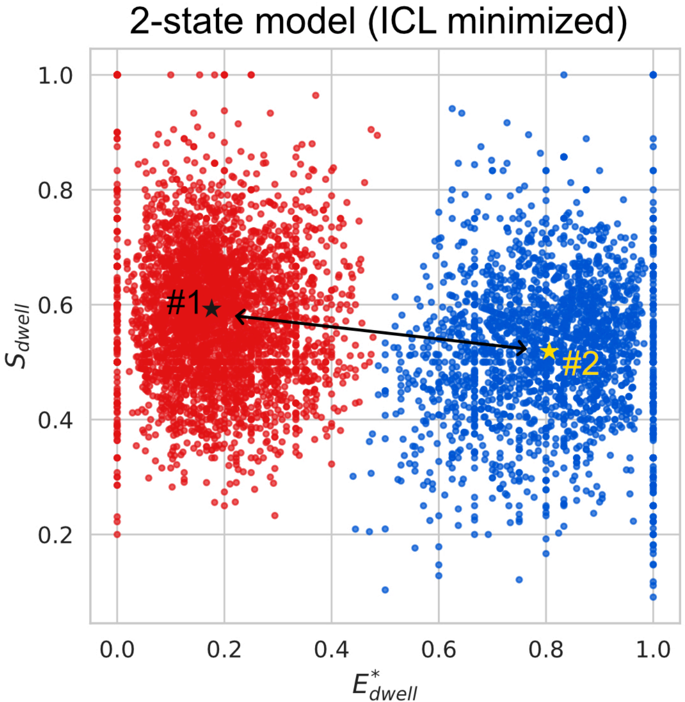

$k_{1\to2}$ = 205.82 /sec
$k_{2\to1}$ = 419.98 /sec

+Ssa1^{ATP}+Ydj1+6AG

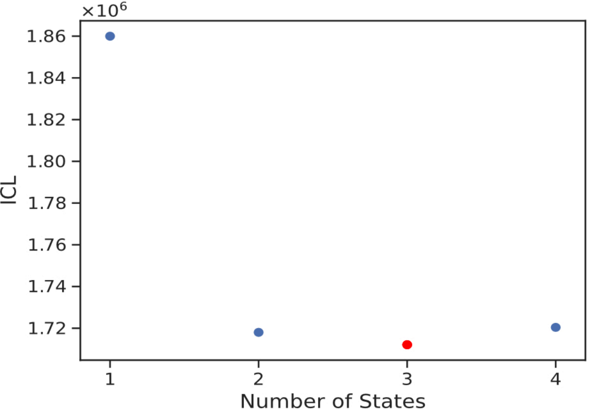

$k_{1\to2}$ = 102.87 ± 9.54 /sec
$k_{1\to3}$ = 1639.97 ± 91.49 /sec
$k_{2\to1}$ = 207.16 ± 12.09 /sec
$k_{2\to3}$ = 150.34 ± 20.06 /sec
$k_{3\to1}$ = 1515.11 ± 81.58 /sec
$k_{3\to2}$ = 76.45 ± 12.88 /sec

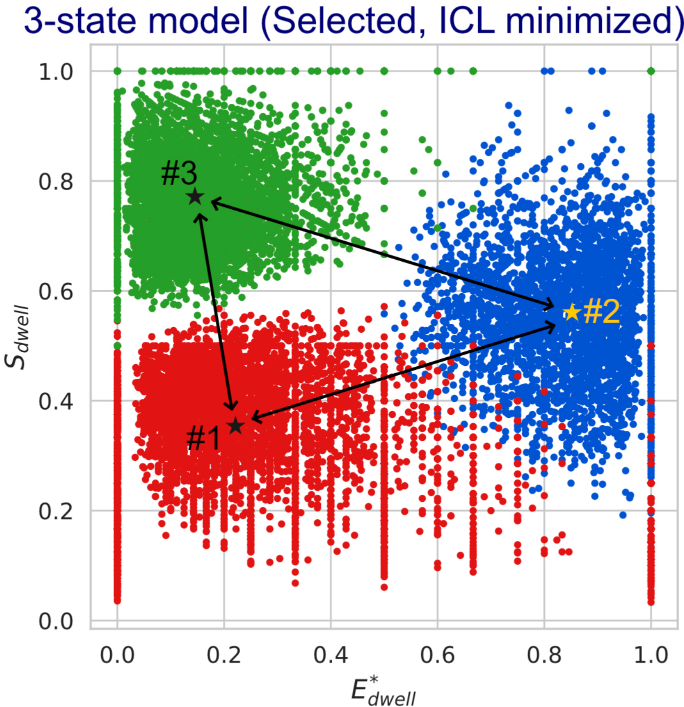

+Ssa1^{ATP}+Ydj1+Bos1+Get3

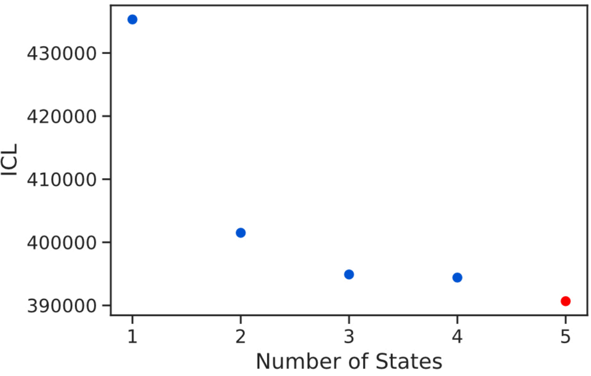

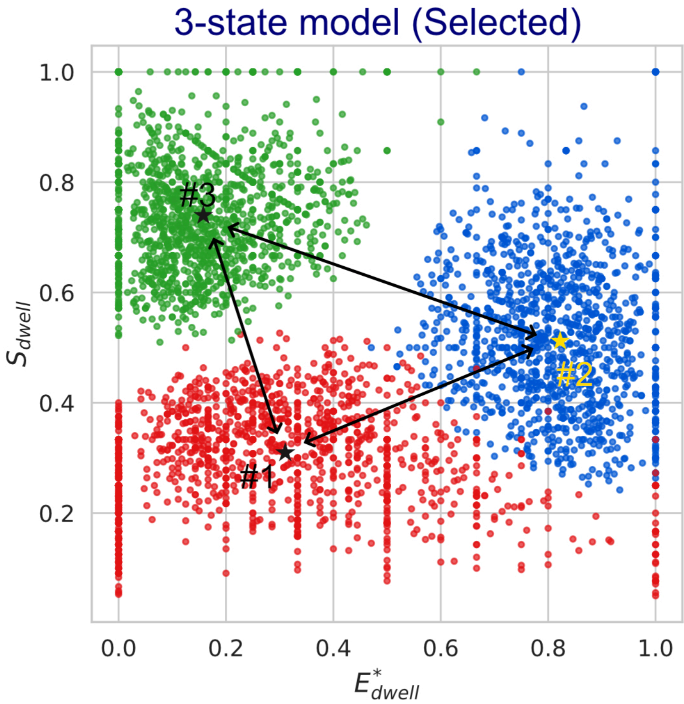

$k_{1\to2}$ = 329.69 /sec
$k_{1\to3}$ = 1385.10 /sec
$k_{2\to1}$ = 372.03 /sec
$k_{2\to3}$ = 270.78 /sec
$k_{3\to1}$ = 1275.68 /sec
$k_{3\to2}$ = 323.97 /sec

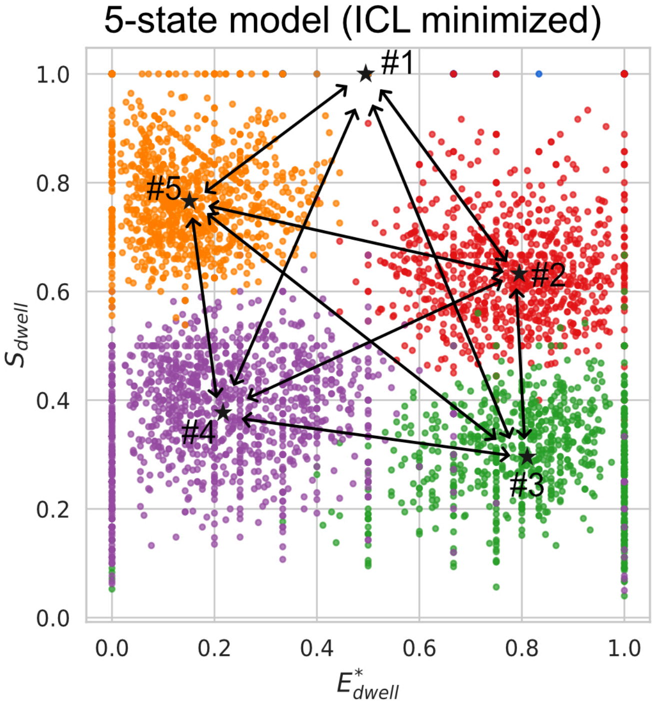

$k_{1\to2}$ = 4967.57 /s
$k_{1\to3}$ = 32033.94 /s
$k_{1\to4}$ = 26170.07 /s
$k_{1\to5}$ = 1.7×10^{-39} /s
$k_{2\to1}$ = 8.60 /s
$k_{2\to3}$ = 826.97 /s
$k_{2\to4}$ = 4.0×10^{-25} /s
$k_{2\to5}$ = 422.83 /s
$k_{3\to1}$ = 3.8×10^{-12} /s
$k_{3\to2}$ = 1314.83 /s
$k_{3\to4}$ = 654.25 /s
$k_{3\to5}$ = 1.2×10^{-21} /s
$k_{4\to1}$ = 3.9×10^{-7} /s
$k_{4\to2}$ = 3.1×10^{-22} /s
$k_{4\to3}$ = 338.94 /s
$k_{4\to5}$ = 1215.76 /s
$k_{5\to1}$ = 1.7×10^{-14} /s
$k_{5\to2}$ = 342.08 /s
$k_{5\to3}$ = 1.9×10^{-62} /s
$k_{5\to4}$ = 1058.69 /s

+Ssa1^{ATP}+Ydj1+Bos1+Get4/5

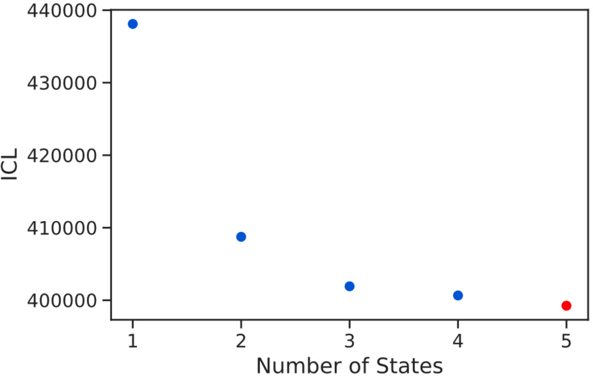

$k_{1\rightarrow2}$ = 414.31 /sec
$k_{1\rightarrow3}$ = 1415.79 /sec
$k_{2\rightarrow1}$ = 406.91 /sec
$k_{2\rightarrow3}$ = 376.64 /sec
$k_{3\rightarrow1}$ = 1414.17 /sec
$k_{3\rightarrow2}$ = 430.64 /sec

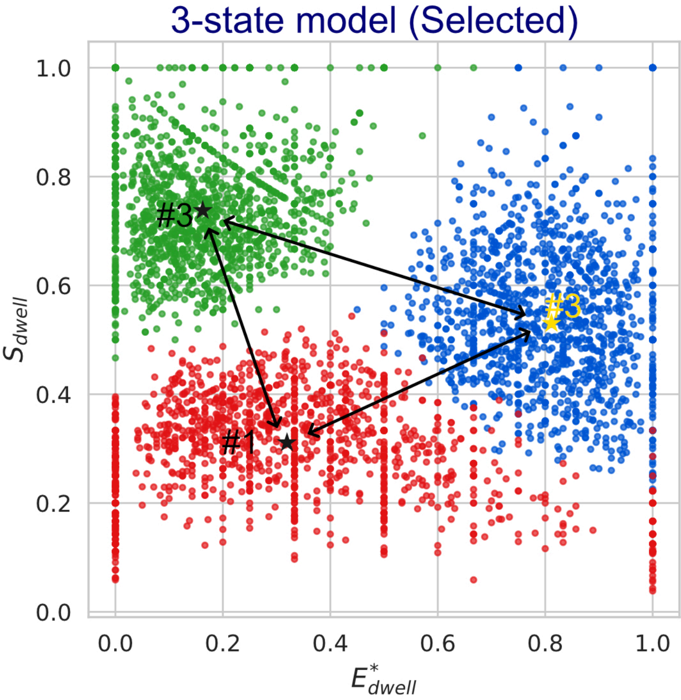

$k_{1\rightarrow2}$ = 1008.41 /s
$k_{1\rightarrow3}$ = 792.20 /s
$k_{1\rightarrow4}$ = 212.12 /s
$k_{1\rightarrow5}$ = 407.77 /s
$k_{2\rightarrow1}$ = 778.22 /s
$k_{2\rightarrow3}$ = 927.03 /s
$k_{2\rightarrow4}$ = 6.0×10^{-16} /s
$k_{2\rightarrow5}$ = 60.23 /s
$k_{3\rightarrow1}$ = 630.87 /s
$k_{3\rightarrow2}$ = 857.43 /s
$k_{3\rightarrow4}$ = 350.91 /s
$k_{3\rightarrow5}$ = 5.3×10^{-7} /s
$k_{4\rightarrow1}$ = 138.76 /s
$k_{4\rightarrow2}$ = 3.7×10^{-20} /s
$k_{4\rightarrow3}$ = 243.42 /s
$k_{4\rightarrow5}$ = 1005.13 /s
$k_{5\rightarrow1}$ = 314.87 /s
$k_{5\rightarrow2}$ = 8.37 /s
$k_{5\rightarrow3}$ = 2.3×10^{-7} /s
$k_{5\rightarrow4}$ = 1368.14 /s

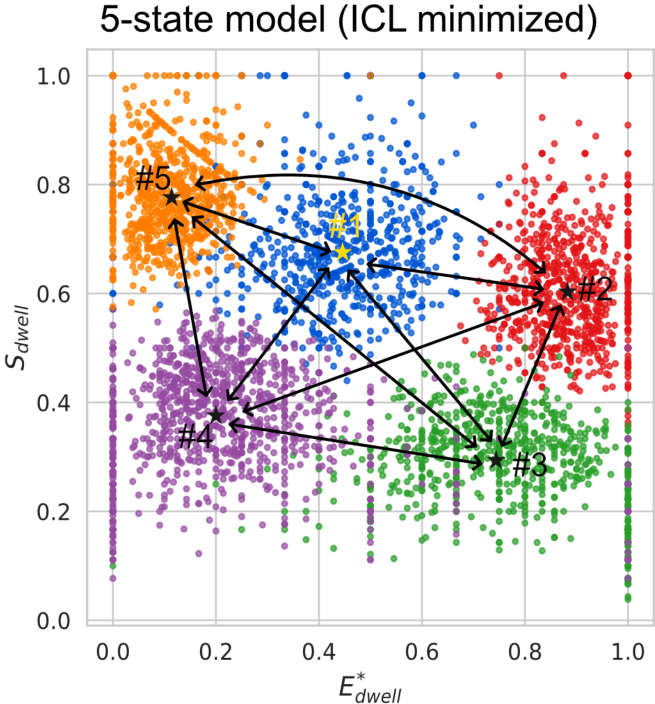

+Ssa1^{ATP}+Ydj1+Bos1+Get3/4/5

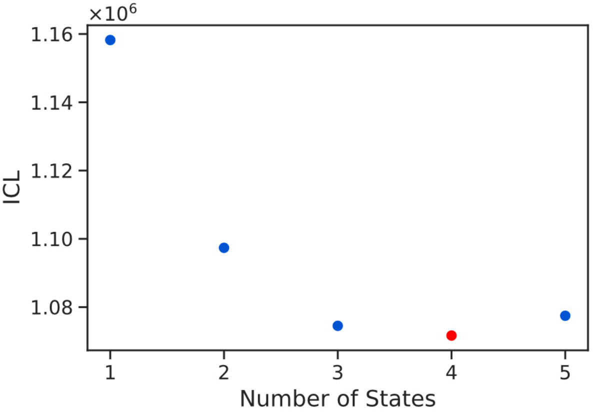

$k_{1\to2}$ = 174.84 ± 54.53 /sec
$k_{1\to3}$ = 1378.01 ± 86.53 /sec
$k_{2\to1}$ = 332.78 ± 61.04 /sec
$k_{2\to3}$ = 429.61 ± 64.51 /sec
$k_{3\to1}$ = 1230.07 ± 77.10 /sec
$k_{3\to2}$ = 236.26 ± 28.20 /sec

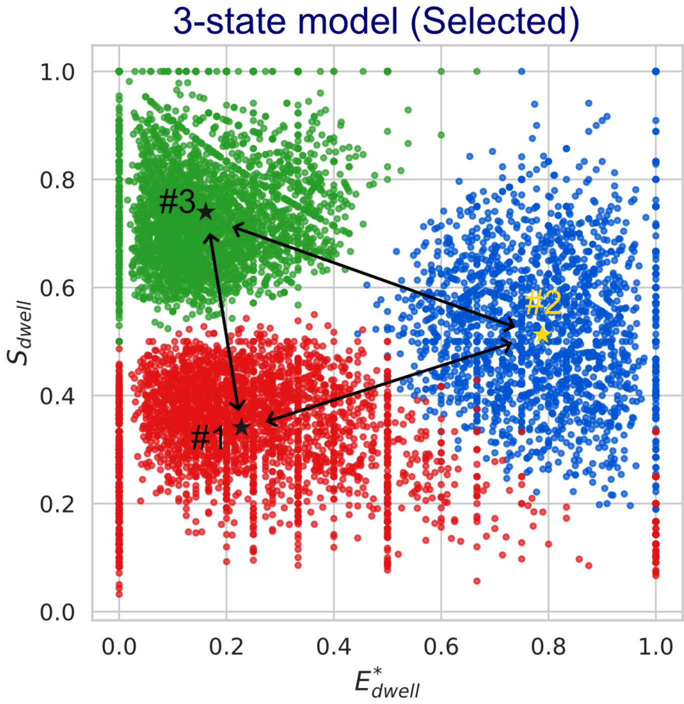

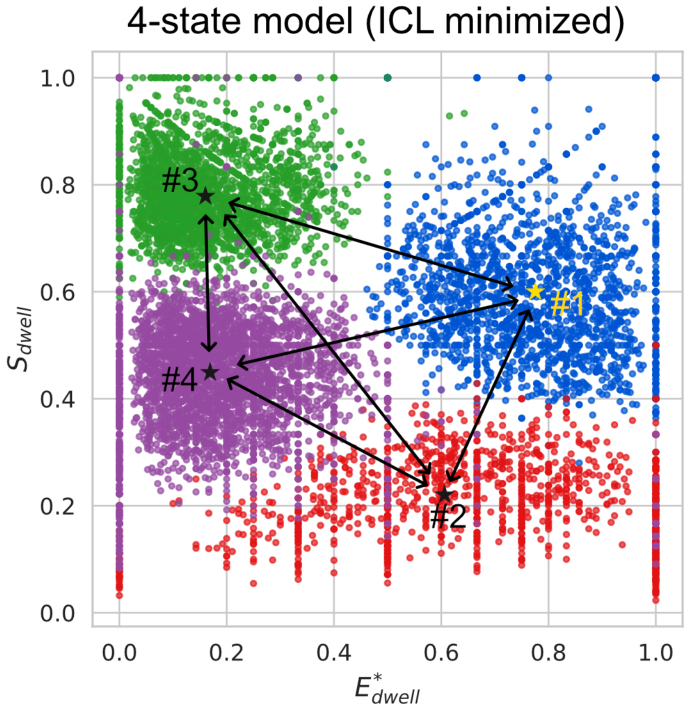

$k_{1\to2}$ = 1222.47 /sec
$k_{1\to3}$ = 525.20 /sec
$k_{1\to4}$ = 3.3×10^{-4} /sec
$k_{2\to1}$ = 1478.95 /sec
$k_{2\to3}$ = 1.9×10^{-64} /sec
$k_{2\to4}$ = 1369.28 /sec
$k_{3\to1}$ = 298.80 /sec
$k_{3\to2}$ = 2.7×10^{-60} /sec
$k_{3\to4}$ = 1185.57 /sec
$k_{4\to1}$ = 4.9×10^{-60} /sec
$k_{4\to2}$ = 487.60 /sec
$k_{4\to3}$ = 960.19 /sec
