# Supplementary material for: Dynamic stability of Sgt2 enables selective and privileged client handover in a chaperone triad
Source: Nat Commun. 2024 Jan 2;15:134. doi: 10.1038/s41467-023-44260-5 (PMC10761869; doi:10.1038/s41467-023-44260-5)

Figure 2a

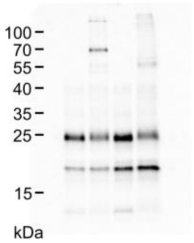

Figure 2b

(one membrane cut in two pieces)

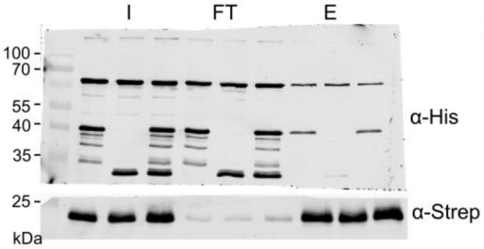

Figure 6e

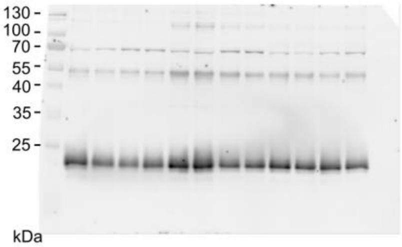

Figure 6f

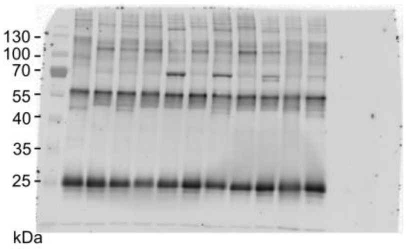

Figure7a

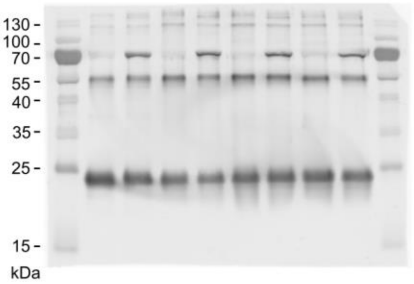

Figure7b

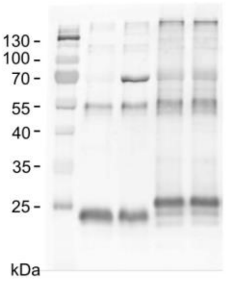

Figure S1c

(one membrane cut in two pieces)

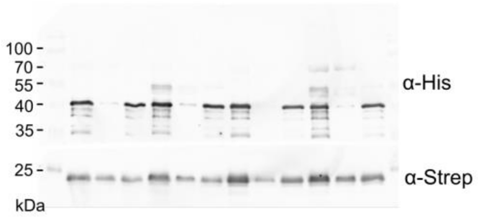

Figure S2a

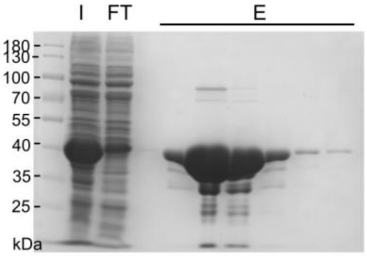

Figure S2b

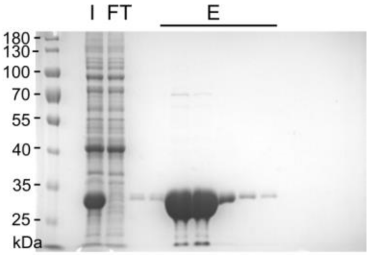

Figure S2c

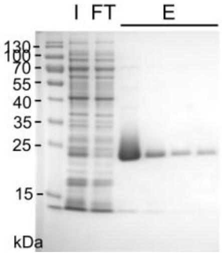

Figure S8d

(one membrane cut in two pieces)

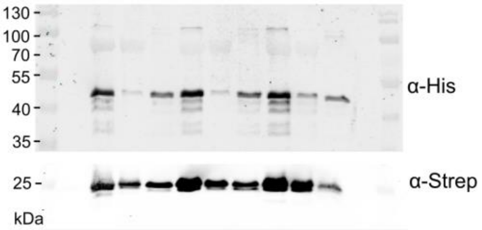

Supplement: Supplementary file 6 — Source data [file 41467_2023_44260_MOESM6_ESM.zip › Source data/Source Data File-uncropped images.pdf]
